# Deep-learning and conventional radiomics to predict *IDH* genotyping status based on magnetic resonance imaging data in adult diffuse glioma

**DOI:** 10.3389/fonc.2023.1143688

**Published:** 2023-08-30

**Authors:** Hongjian Zhang, Xiao Fan, Junxia Zhang, Zhiyuan Wei, Wei Feng, Yifang Hu, Jiaying Ni, Fushen Yao, Gaoxin Zhou, Cheng Wan, Xin Zhang, Junjie Wang, Yun Liu, Yongping You, Yun Yu

**Affiliations:** ^1^ Department of Medical Informatics, School of Biomedical Engineering and Informatics, Nanjing Medical University, Nanjing, Jiangsu, China; ^2^ Department of Neurosurgery, The First Affiliated Hospital of Nanjing Medical University, Nanjing, Jiangsu, China; ^3^ Department of Geriatric Endocrinology, The First Affiliated Hospital of Nanjing Medical University, Nanjing, Jiangsu, China; ^4^ Institute of Medical Informatics and Management, Nanjing Medical University, Nanjing, Jiangsu, China

**Keywords:** IDH, glioma, deep-learning, radiomics, magnetic resonance imaging

## Abstract

**Objectives:**

In adult diffuse glioma, preoperative detection of isocitrate dehydrogenase (*IDH*) status helps clinicians develop surgical strategies and evaluate patient prognosis. Here, we aim to identify an optimal machine-learning model for prediction of *IDH* genotyping by combining deep-learning (DL) signatures and conventional radiomics (CR) features as model predictors.

**Methods:**

In this study, a total of 486 patients with adult diffuse gliomas were retrospectively collected from our medical center (n=268) and the public database (TCGA, n=218). All included patients were randomly divided into the training and validation sets by using nested 10-fold cross-validation. A total of 6,736 CR features were extracted from four MRI modalities in each patient, namely T1WI, T1CE, T2WI, and FLAIR. The LASSO algorithm was performed for CR feature selection. In each MRI modality, we applied a CNN+LSTM–based neural network to extract DL features and integrate these features into a DL signature after the fully connected layer with sigmoid activation. Eight classic machine-learning models were analyzed and compared in terms of their prediction performance and stability in *IDH* genotyping by combining the LASSO–selected CR features and integrated DL signatures as model predictors. In the validation sets, the prediction performance was evaluated by using accuracy and the area under the curve (AUC) of the receiver operating characteristics, while the model stability was analyzed by using the relative standard deviation of the AUC (RSD_AUC_). Subgroup analyses of DL signatures and CR features were also individually conducted to explore their independent prediction values.

**Results:**

Logistic regression (LR) achieved favorable prediction performance (AUC: 0.920 ± 0.043, accuracy: 0.843 ± 0.044), whereas support vector machine with the linear kernel (l-SVM) displayed low prediction performance (AUC: 0.812 ± 0.052, accuracy: 0.821 ± 0.050). With regard to stability, LR also showed high robustness against data perturbation (RSD_AUC_: 4.7%). Subgroup analyses showed that DL signatures outperformed CR features (DL, AUC: 0.915 ± 0.054, accuracy: 0.835 ± 0.061, RSD_AUC_: 5.9%; CR, AUC: 0.830 ± 0.066, accuracy: 0.771 ± 0.051, RSD_AUC_: 8.0%), while DL and DL+CR achieved similar prediction results.

**Conclusion:**

In *IDH* genotyping, LR is a promising machine-learning classification model. Compared with CR features, DL signatures exhibit markedly superior prediction values and discriminative capability.

## 1 Introduction

Adult diffuse gliomas are a group of primary malignant brain tumors and have a relatively high mortality rate (1). Despite the availability of a diverse array of treatments including tumor resection, radiotherapy, chemotherapy, and experimental targeted therapy, the prognosis for patients remains generally unfavorable (2, 3). According to the 2007 World Health Organization Central Nervous System (2007 WHO CNS) tumor classification (4), adult diffuse gliomas are classified based on tumor histology. However, a growing number of studies have shown that adult diffuse gliomas with different histological classifications may have similar biological behaviors and prognosis because of the same genetic changes (5). Therefore, the two newest guidelines from the WHO CNS and European Association of Neuro-Oncology (EANO), both published in 2021, underscore the significance of incorporating molecular biomarkers with both clinical and pathological values into the precision classification of adult diffuse gliomas, to promote the development of tumor precision treatment (6, 7).

One of the important molecular biomarkers for adult diffuse gliomas is the expression status of isocitrate dehydrogenase (*IDH*), which is now routinely incorporated into the clinical management of patients (7). According to the 2021 EANO guideline for adult diffuse gliomas, the presence of an *IDH* mutation can be diagnosed as an astrocytoma with WHO grade 2–4 or an oligodendroglioma with WHO grade 2–3. Conversely, in most cases, the absence of an *IDH* mutation indicates the diagnosis of a glioblastoma with WHO grade 4. Some studies have shown that adult patients with *IDH*-wildtype glioblastoma undergoing standard treatment typically have an average overall survival of 15–18 months (8), while the average overall survival can extend up to 14.7 years in cases of oligodendroglioma with *IDH* mutation and *1p/19q*-codeletion, but with appropriate treatment (9, 10). Furthermore, the presence of *IDH* mutation has emerged as a specific treatment target, leading to its exploration in various clinical trials involving peptide vaccination and small-molecule inhibitor approaches (11, 12). Therefore, the *IDH* expression status is highly relevant to the glioma prognosis and keeps high clinical value for the classification of adult diffuse gliomas.

In clinical practice, *IDH* genotyping is carried out on biopsied tumor samples. The limitation of this approach is the relatively long detection period, invasiveness, and the sampling difficulty from certain brain areas. Conversely, magnetic resonance imaging (MRI) has been considered the most promising candidate to aid decision-making in clinical practice due to its non-invasive nature, fast and global detection ability, and high resolution for soft tissues (13, 14). Taking full advantage of the wealth of information obtained from preoperative MRIs could assist in filling the knowledge gaps in local tumor biopsy. Thus, MRI–based *IDH* genotyping appears to be an important preoperative assessment method to help guide the treatment and prognosis of glioma patients.

Radiomics (15), an imaging analysis method, advocates that the high-throughput quantitative handcrafted features extracted from medical images can be utilized to build machine-learning models to enable the preoperative evaluation of tumors. Several studies have investigated the potential of MRI–based radiomics analysis to noninvasively facilitate tumor grading, molecular subtyping, and prognosis evaluation in gliomas (16–19). However, in some aspects, conventional radiomics (CR) involves rigorous and complex analysis to inevitably require extracting and selecting handcrafted features that could introduce additional errors because of feature calculations (20). Furthermore, the limited and handcrafted radiomics features cannot adequately reflect tumor heterogeneities, which could limit the prediction abilities of machine-learning models (21).

Nowadays, advances in computational technology have promoted the development of deep-learning (DL) that has been widely used in tumor preoperative evaluation given its end-to-end prediction advantage and ability to simplify the analysis process (22, 23). In contrast to CR, deep neural networks possess remarkable representation capabilities, enabling the extraction of high-throughput discriminative features that can directly capture abundant tumor information. These deep neural networks will eliminate the need for additional feature extraction and selection operations, simplifying the process while still yielding valuable insights. DL features can further reveal tumor heterogeneity and stimulate the prediction potential of machine-learning models. In glioma *IDH* genotyping, machine-learning models only based on CR features have been widely applied and analyzed (24, 25). However, little work has been done on evaluating the effectiveness of different machine-learning models by combining DL signatures and CR features as model predictors.

In the present study, we aimed to determine the optimal machine-learning model by making full use of the DL signatures and CR features. We built an image sequence model to extract DL signatures. Based on the extracted DL signatures and CR features from four conventional MRI modalities used in most medical centers, we evaluated and compared eight classic machine-learning models in terms of their stability and prediction performance in *IDH* genotyping. Furthermore, the subgroup analyses of DL signatures and CR features were also individually conducted to explore their independent prediction values.

## 2 Materials and methods

### 2.1 Patient enrollment

All the cases used in this retrospective study were de-identified and obtained from the public datasets (TCGA-GBM, TCGA-LGG), and our local medical center (The First Affiliated Hospital of Nanjing Medical University) between May 2015 and July 2020. Institutional Review Board approval from our medical center was obtained, but this was not required for the public datasets. Patients who met the following criteria were included in this study: (1) pathological diagnosis of primary diffuse glioma; (2) age≥18 years; (3) known *IDH* status (detected by immunohistochemistry or Sanger sequencing); (4) no history of preoperative therapy, biopsy, or any treatment; and (5) four available preoperative MRI modalities, including T1-weighted, T2-weighted, gadolinium contrast-enhanced T1-weighted, and T2-weighted fluid-attenuated inversion recovery images (T1WI, T2WI, T1CE, and FLAIR, respectively).

### 2.2 MR imaging

The MR images are heterogeneous, because they are acquired using either 1.5T or 3.0T MRI scanners according to the different imaging protocols at each institution. The MRI scanners were provided by different MR vendors, including Philips, General Electric, and Siemens. The preoperative MR imaging protocols include the acquisition of T1WI (parameters vary from TR: 300–2000 ms, TE: 5–25 ms, FOV: 60–100 mm, slice thickness: 1.5–7.5 mm, and matrix: 256×256 or 512×512); T2WI (parameters vary from TR: 2000–10000 ms, TE: 80–150 ms, FOV: 70–110 mm, slice thickness: 1.5–8 mm, and matrix: 256×256 or 512×512); T1CE (parameters vary from TR: 200–1100 ms, TE: 4–20 ms, FOV: 70–100 mm, slice thickness: 1–8.5 mm, and matrix: 256×256 or 512×512); and FLAIR (parameters vary from TR: 5000–11000 ms, TE: 80–200 ms, FOV: 70–110 mm, slice thickness: 2–7 mm, and matrix: 256×256 or 512×512). All MR imaging data including pixel matrixes and metadata were saved in the digital imaging and communications in medicine (DICOM) format.

### 2.3 Image preprocessing and tumor segmentation

We converted MR images with DICOM format into the neuroimaging informatics technology initiative (NIFTI) format by using the python package SimpleITK. Referring to the image preprocessing in the competition of brain tumor segmentation (BraTS2021) (https://www.med.upenn.edu/cbica/brats2021), images were co-registered to the same anatomical template (SRI24) (26), interpolated to a uniform isotropic resolution (1mm^3^) and skull-stripped by using FSL software (https://fsl.fmrib.ox.ac.uk/fsl).

Tumor segmentation is a crucial step for the following feature extraction and quantitative analysis. As is known in the BraTS competition, we can split gliomas into two subregions: tumor core (TC, comprising a contrast-enhancing area and necrotic portions, if any) and the whole tumor (WT, combining the tumor core and edema). TC describes the bulk of the tumor, which is what is typically resected, while WT describes the complete extent of the disease. In our study, we used the fully automated nnU-Net segmentation framework based on a convolutional neural network (CNN) to segment these two tumor subregions. The nnU-Net framework is the first plug-and-play tool for biomedical image segmentation (https://github.com/MIC-DKFZ/nnUNet). It has been widely validated in the BraTS competition and achieved superior performance in brain tumor segmentation (27). Inexperienced users can use nnU-Net out of the box for their custom 3D segmentation problem without the need for manual intervention.

### 2.4 DL signature extraction

**Figure 1:**
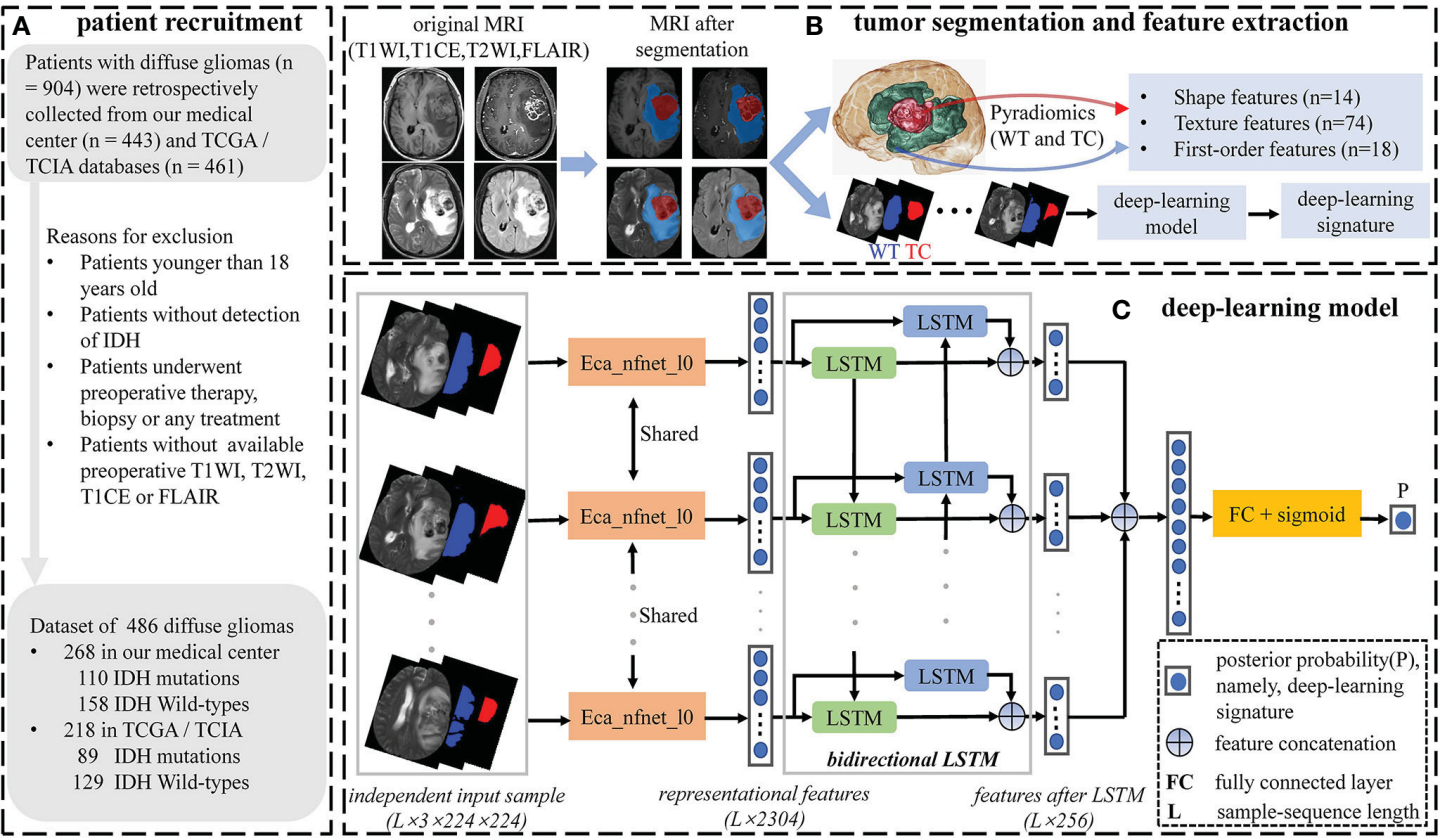
Workflow of patient recruitment, tumor segmentation and feature selection. **(A)** Patient recruitment process. **(B)** Tumor segmentation and feature extraction process. Tumor segmentation was performed with T1WI, T1CE, T2WI, and FLAIR images by using nnU-Net automated segmentation framework. Conventional radiomics features were extracted from the WT and TC volumes under every single-modality MRI, respectively. **(C)** The deep-learning model. Single-modality DL signature was extracted using the deep-learning model.

Convolutional neural networks and recurrent neural networks (RNN) are different types of artificial neural networks that can perform representational learning on imaging data and provide different hierarchical feature representations at each network layer (28). It is precisely the stacking employment of multiple network layers with non-linear activation functions that make the feature representation complex and diverse. After passing through a series of chained CNN or RNN layers, posterior probability can be calculated from the representational features and used as a predictor for tumor preoperative evaluation. In our study, we combined CNN–based eca_nfnet_l0 (29) and RNN–based long-short-term memory network (LSTM) (30) together as our DL model, extracting posterior probability as the DL signature for *IDH* genotyping (**Figure 1**).

Data preparation needs to be conducted before the imaging data is imported into the DL model. To avoid heterogeneity bias, various image signal intensities were transformed into standardized intensity ranges via z-score normalization (*Z* = (*x*-*μ*)/*σ*), where *μ* and *σ* are the mean and standard deviation of pixel values, respectively). Typically, glioma volumes can reflect abundant and complex tumor characteristics under different spatial dimensions. To utilize abundant tumor information from three spatial dimensions, we extracted the axial, coronal, and sagittal tumor slices from each 3D image. Referring to the segmented WT mask, we selected slices with the largest tumor area from each spatial dimension, as well as slices with tumor regions of 50th, 55th, 60th, 65th, 70th, 75th,80th, 85th, 90th, and 95th percentiles. Subsequently, we integrated each selected slice with its two corresponding mask slices (WT and TC) to create a new 3-channel image. A total of 33 representative 3-channel images were created from each 3D image. Unlike conventional 2D-CNN models, our model takes the correlation between different tumor slices as well as the hidden tumor spatial information into account. When it comes to extracting features using LSTM, we need to feed an image sequence representing an independent input sample into the model. Thereupon, all 3-channel images needed to be arranged according to their respective slice ordinal numbers and integrated into an image sequence based on the order of axial, coronal, and sagittal sequences. Finally, in each 3D image, we obtained the independent input samples with a total number of ((33-length)/stride+1) from the 3-channel image sequence with a total length of 33 by specifying the sample-sequence length and moving stride (**Supplementary Figure S1**). We also performed random flip, rotation, and translation on the independent samples to enhance the robustness of the DL model, as well as resampled to 224×224×3 (determined by the pretrained ImageNet dataset (31)).

Eca_nfnet_l0 is a variant of the nfnet (normalization free net) model family, whose prediction performance achieved on the ImageNet dataset is better than that of the residual networks (ResNet) (31). We utilized eca_nfnet_l0 to obtain the representational features from each 3-channel image of the independent input sample. Bidirectional LSTM was then used to learn the intrinsic and mutual relationships of the representational features from different 3-channel images. Finally, the output features of LSTM were ensembled into a class posterior probability (namely, DL signature) through a fully connected layer with a sigmoid activation function. A single patient will have multiple independent input samples under every single-modality MRI. We considered the average of all posterior probabilities from different input samples as the patient’s final DL signature. A total of four DL signatures were independently extracted from four single-modality MRIs.

### 2.5 Conventional radiomics feature extraction

To reduce heterogeneity bias between patients, MR images were normalized via z-score normalization for subsequent CR feature extraction, which was based entirely on an open-source python package pyradiomics that was established to provide a reference standard according to the image biomarker standardization initiative (IBSI) for radiomics analysis of medical imaging (32). Based on the segmented sub-volumes (WT and TC), we extracted shape, first-order, and texture features from original and derived images (wavelet decompositions via directional low-pass and high-pass filtering to yield eight derived images on each original 3D MR image).

Shape features describe the size and shape of the region of interest (ROI). These features are independent of the gray-level intensity distribution in the ROI and are only calculated on 3D mask. First-order features take the properties of individual pixel values ignoring the spatial interaction between image pixels into account. While texture features describe this spatial interaction between every image pixel and their surrounding neighborhoods. Texture features can be extracted using five methods, including the gray-level co-occurrence matrix (GLCM), gray-level run length matrix (GLRLM), gray-level size zone matrix (GLSZM), neighborhood gray-tone difference matrix (NGTDM), and gray-level dependence matrix (GLDM).

### 2.6 Feature selection and model development

#### 2.6.1 Nested-CV

**Figure 2:**
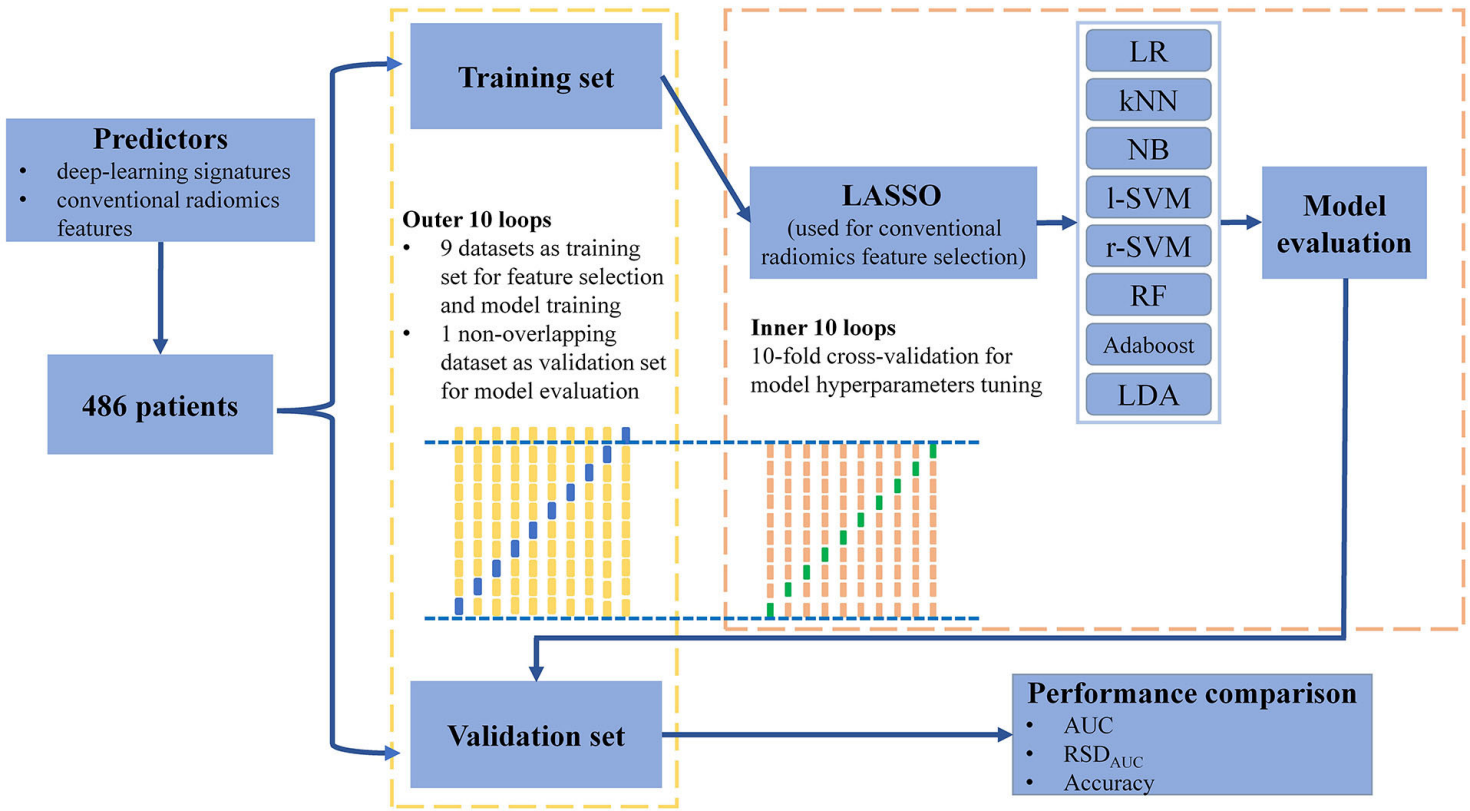
Workflow of machine-learning method training and validation.

Nested cross-validation (nested-CV) is a search for hyperparameters by estimating the generalization error of the training model to obtain the best hyperparameters. It consists of the outer loops and inner loops. The inner loop refers to the cross-validation with the ability to search for the best hyperparameters to provide the best hyperparameters for the training model validated in the outer loop. The outer loop provides training data to the inner loop while retaining some extra data to validate the model trained in the inner loop. Compared to simple-CV, nested-CV can prevent information leakage of data to obtain a relatively low model scoring bias, especially in relatively small datasets. Nested-CV is also successfully employed in the machine-learning analysis of neuroimaging (33). In the present study, we utilized nested-10-fold-CV to perform feature selection and model hyperparameters tuning, in which nine non-overlapping datasets of each outer loop were trained in its inner loop and the remaining one non-overlapping dataset was the validation set of this outer loop (**Figure 2** and **Supplementary Table S1**).

#### 2.6.2 Conventional radiomics feature selection

When building a machine-learning classifier involving high-throughput features, feature selection provides a crucial step to reduce the risk of over-fitting, improve accuracy, and decrease training time. Before performing CR feature selection, all CR features are subjected to z-score normalization to eliminate the influence of different feature magnitudes. We utilized the least absolute shrinkage and selection operator penalty (LASSO) algorithm with L1 regularization to select CR features (34). A hyperparameter, designated as *α*, controls the extent of L1 regularization: the larger the value of , the fewer the selected CR features. The optimal hyperparameter *α*, ranging from 0 to 0.1 in 0.05 increments, was selected by using the inner 10-fold cross-validation in the nine non-overlapping datasets of each outer loop (33). We then retained the CR features with a non-zero coefficient resulting from the optimal *α* for further analyses. In this way, 10 specific CR feature subsets were selected by using the LASSO model with optimal *α*.

#### 2.6.3 Classifier building

After the CR feature selection, we utilized the mixture of DL signatures and CR features as predictors to construct machine-learning classifiers. Eight classical machine-learning classifiers were built and compared: logistic regression (LR), k-nearest neighbors (kNN), naive Bayes (NB), support vector machines with the linear kernel (l-SVM), support vector machines with radial basis function kernel (r-SVM), random forest (RF), adaptive boosting (Adaboost) and linear discriminant analysis (LDA) (**Supplementary Table S2**). In each outer loop, we tuned every classifier by using the inner 10-fold cross-validation and compared the area under the curve (AUC) value of the receiver operating characteristics to identify the classifier with optimal hyperparameters. Random grid searches were used for all hyperparameter tuning processes. The average prediction performance of classifiers was estimated in the validation sets of the outer loops by quantifying the accuracy and AUC values. Meanwhile, the stability of the machine-learning classifier was quantified by using the relative standard deviation of the AUC value (RSD_AUC_). *RSD_AUC_*% is defined as:


$$RSD_{AUC}\%=\frac{\sigma_{AUC}}{\mu_{AUC}}\times 100\%$$

Where, *σ_AUC_* and *μ_AUC_* are the standard deviation and mean of the AUC value, respectively. A lower *RSD_AUC_*% value represents the higher stability of the machine-learning classifier.

### 2.7 Implementation details

All the calculations and modeling were based on the pytorch, sklearn, and pycaret libraries in python as the backend. When building the DL model, we created consecutive image sequences as the independent input samples by setting the sample-sequence length to 11 and moving stride to 2. In this way, we could obtain 12 independent samples from single patient under every single-modality data. We used the sigmoid linear unit activation function in each hidden layer and the binary cross-entropy as the objective function. The weights of the network were optimized via a gradient descent algorithm with a mini-batch size of 32 and a learning rate of 1e-6.

## 3 Results

### 3.1 Patient and tumor characteristics

Overall, we obtained 486 cases and the patient and tumor characteristics were statistically analyzed and shown in **Table 1**. The workflow of the current study is displayed in **Figures 1** and **2**. The proportions of patients with *IDH* mutation and wildtype were 41% (199/486) and 59% (287/486), respectively. There was no significant difference in sex between patients with *IDH* mutation and wildtype (p>0.05). The proportion of patients presenting with LGG was 89% (177/199) in *IDH* mutation patients, while the proportion of patients presenting with HGG was 78% (224/287) in *IDH* wildtype patients (p<0.05). The mean (± standard deviation) age was 51.7 ± 14.2 years and its P-value was<0.05 between patients with *IDH* mutation and *IDH* wildtype.

**Table 1:** Patient and tumor characteristics of the entire cohort.

Parameters	Entire cohort	Public dataset		P_intra_	Local population		P_intra_	P_inter_
		IDH expression status			IDH expression status			
		Mutation	Wildtype		Mutation	Wildtype		
Total Number	486	89	129		110	158		
Sex				0.24			0.79	0.32
Male	274(56%)	43(48%)	74(57%)		66(60%)	91(58%)		
Female	212(44%)	46(52%)	55(43%)		44(40%)	67(42%)		
Grade				<0.05			<0.05	0.83
LGG(WHO 2 or 3)	240(49%)	84(94%)	22(17%)		93(85%)	41(26%)		
HGG(WHO 4)	246(51%)	5(6%)	107(83%)		17(15%)	117(74%)		
Age (years)	51.7 ± 14.2	43.9 ± 13.9	58.3 ± 13.0	<0.05	44.3 ± 11.7	55.8 ± 12.6	<0.05	1.01
18 - 51	232(48%), 39.6 ± 8.8	62(70%), 36.6 ± 8.9	34(26%), 41.8 ± 9.0		82(75%), 39.2 ± 8.4	54(34%), 42.1 ± 8.1		
≥ 52	254(52%), 62.8 ± 7.8	27(30%), 60.7 ± 6.9	95(74%), 64.2 ± 8.3		28(25%), 59.3 ± 5.1	104(66%), 62.9 ± 7.7		
Histology				<0.05			<0.05	0.57
Astrocytoma	118(24%)	22(25%)	10(8%)		53(48%)	33(21%)		
Oligodendroglioma	92(19%)	40(45%)	8(6%)		39(36%)	5(3%)		
Oligoastrocytoma	39(8%)	22(25%)	4(3%)		10(9%)	3(2%)		
Glioblastoma	237(49%)	5(5%)	107(83%)		8(7%)	117(74%)		
1p/19q				<0.05			<0.05	0.92
Codeletion	66(14%)	21(24%)	0(0%)		37(34%)	8(5%)		
Nono-codeletion	240(49%)	40(45%)	22(17%)		58(53%)	120(76%)		
Unknown	180(37%)	28(31%)	107(83%)		15(13%)	30(19%)		
MGMT				0.09			0.35	0.14
Methylation	110(22%)	5(6%)	27(21%)		32(29%)	46(29%)		
Unmethylation	139(29%)	0(0%)	28(22%)		37(34%)	74(47%)		
Unknown	237(49%)	84(94%)	74(57%)		41(37%)	38(24%)		

Data is presented as the mean ± standard deviation or as numbers (with percentage in parentheses). Age is divided into two groups based on its mean value. P-values are calculated from unpaired t-test for continuous variables and chi-square test for categorical variables, to compare statistical differences of variables between IDH mutation and IDH wildtype (P_intra_) as well as between public and local cohorts (P_inter_). A P<0.05 is considered to indicate statistically significant differences.

### 3.2 Conventional radiomics features and DL signatures

In each MR modality, we extracted 842 CR features from each tumor subregion, respectively, including 18×9 first-order, 23×9 GLCM, 16×9 GLRLM, 16×9 GLSZM, 5×9 NGTDM, 14×9 GLDM, and 14 shape features. A total of 6,736 CR features were extracted from the MRI data of every patient. After LASSO, 10 specific CR feature subsets were obtained, whose numbers ranged from 5 to 9 (**Supplementary Table S3**). Features that were selected in at least five of the 10 loops were considered the most valuable and stable CR features (33). Finally, we obtained seven valuable CR features, comprising texture, first-order, and shape features extracted from the original images and wavelet-transformed images. These seven CR features together with four DL signatures were then compared with the *IDH* mutation status by using the unpaired *t*-test, revealing that all the selected features were significantly different between patients with *IDH* mutation and wildtype (p<0.05) (**Supplementary Table S4**).

### 3.3 Model performance

#### 3.3.1 Classifiers using DL+CR features as predictors

We analyzed the prediction performance and stability of the eight classic machine-learning classifiers by using the DL+CR features as predictors (**Table 2**). In terms of the prediction performance in all classifiers, LR had the best AUC (0.920 ± 0.043), r-SVM had the best accuracy (0.856 ± 0.056), while l-SVM had the lowest prediction performance with AUC (0.812 ± 0.052) and accuracy (0.821 ± 0.050). In terms of stability, the most stable classifier was LR (RSD_AUC_: 4.7%), followed by LDA (RSD_AUC_: 4.9%) and Adaboost (RSD_AUC_: 4.9%). kNN (RSD_AUC_: 9.0%) and r-SVM (RSD_AUC_: 8.0%) had the lowest stability among all the classifiers. We mainly referred to the AUC value to compare the prediction performance between different classifiers. Overall, LR with the best AUC and stability (AUC: 0.920 ± 0.043, RSD_AUC_: 4.7%) outperformed other machine-learning classifiers in the *IDH* genotyping prediction.

**Table 2:** Validation performance based on DL+CR features.

Classifier	Accuracy	AUC	RSD_AUC_
LR	0.843 ± 0.044	**0.920 ± 0.043**	**4.7%**
kNN	0.848 ± 0.065	0.904 ± 0.081	9.0%
NB	0.850 ± 0.052	0.916 ± 0.063	6.9%
l-SVM	0.821 ± 0.050	0.812 ± 0.052	6.4%
r-SVM	**0.856 ± 0.056**	0.911 ± 0.073	8.0%
RF	0.842 ± 0.051	0.917 ± 0.052	5.7%
Adaboost	0.827 ± 0.050	0.903 ± 0.044	4.9%
LDA	0.839 ± 0.048	0.918 ± 0.045	4.9%
Average	0.841 ± 0.051	0.900 ± 0.065	7.2%

Numbers in bold font represent the best performance among different models.

#### 3.3.2 The optimal classifier using different feature subcategories as predictors

The DL signatures and CR features were individually analyzed to investigate their respective prediction potential in *IDH* genotyping. The average prediction performance of the DL signatures among the eight classifiers (AUC: 0.900 ± 0.058, accuracy: 0.835 ± 0.056, RSD_AUC_: 6.4%) was better than that of the CR features (AUC: 0.799 ± 0.095, accuracy: 0.754 ± 0.072, RSD_AUC_: 11.9%). In contrast, the average prediction performance of the DL+CR features had little improvement (AUC: 0.900 ± 0.065, accuracy: 0.841 ± 0.051, RSD_AUC_: 7.2%) (**Table 3** and **Supplementary Table S5**).

**Table 3:** Average validation performance of different feature subcategories.

Subcategory	Accuracy	AUC	RSD_AUC_
CR	0.754 ± 0.072	0.799 ± 0.095	11.9%
DL	0.835 ± 0.056	**0.900 ± 0.058**	**6.4%**
DL+CR	**0.841 ± 0.051**	**0.900 ± 0.065**	7.2%

Numbers in bold font represent the best performance among different models.

**Figure 3:**
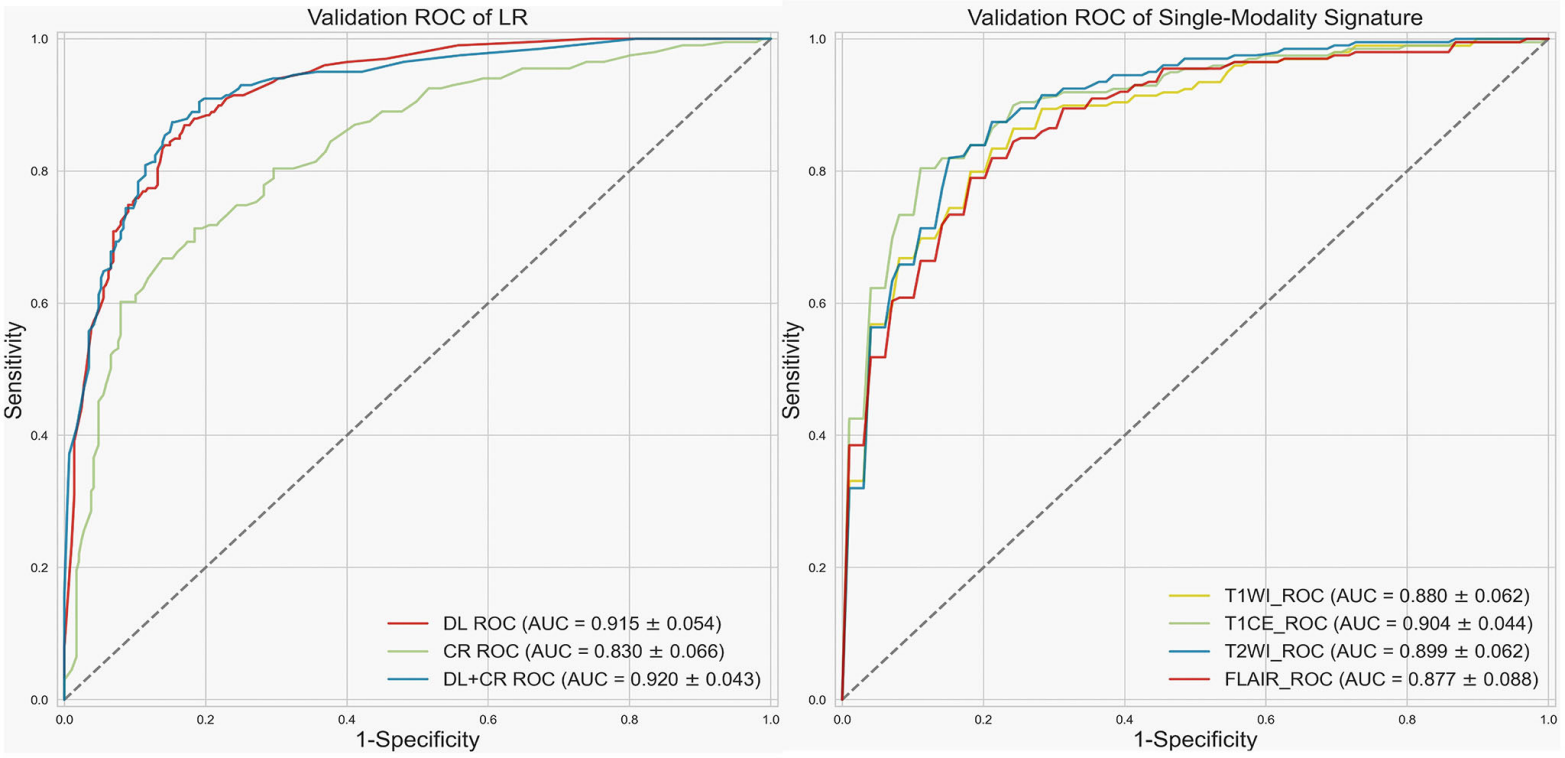
Receiver operating characteristic (ROC) curve. Left, validation ROC curve of LR by using different feature subcategories as predictors. Right, validation ROC curve for single-modality signature.

**Figure 4:**
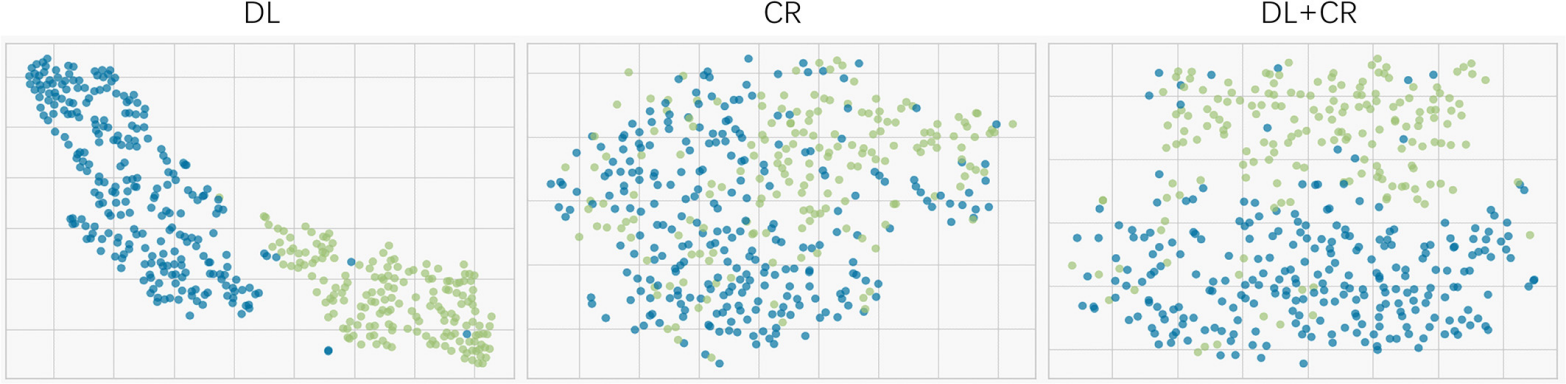
t-SNE visualization for different feature subcategories (the DL, CR and DL+CR). Every dot represents a patient. Blue represents the patients with *IDH* wildtype, whereas green represents the patients with IDH mutation.

Since LR had the best prediction performance and stability in DL+CR, we additionally utilized it to compare the prediction potential of the different feature subcategories. As illustrated in **Figure 3**, LR in the DL achieved the favorable prediction results and stability (AUC: 0.915 ± 0.054, accuracy: 0.835 ± 0.061, RSD_AUC_: 5.9%). In contrast, LR in the CR achieved the lowest prediction results and stability (AUC: 0.830 ± 0.066, accuracy: 0.771 ± 0.051, RSD_AUC_: 8.0%). After combining the DL signatures and CR features, we found that the CR features did not obviously help to improve the prediction performance of LR (AUC: 0.920 ± 0.043, accuracy: 0.843 ± 0.044, RSD_AUC_: 4.7%). Furthermore, we analyzed the prediction potential of single-modality DL signature in *IDH* genotyping by ROC analysis. The T1CE signature had the best AUC (AUC: 0.904 ± 0.044, RSD_AUC_: 4.9%), followed by the T2WI signature (AUC: 0.899 ± 0.062, RSD_AUC_: 6.9%), T1WI signature (AUC: 0.880 ± 0.062, RSD_AUC_: 7.0%) and FLAIR signature (AUC: 0.877 ± 0.088, RSD_AUC_: 10.0%). The *t*-SNE visualization of different feature subcategories are also shown in **Figure 4**, illustrating that the DL signatures possess better discriminative ability than the CR features.

## 4 Discussion

According to the newest WHO classification of CNS tumors, adult diffuse gliomas were classified not only by pathological characteristics but also by genotyping, which was the important prognostic factor affecting patients’ survival. In addition to *IDH* genotyping, genetic phenotypes and molecular characteristics such as chromosome *1p/19q* co-deletion, O-6-methylguanine-DNA methyltransferase (*MGMT*) methylation, and phosphatase and tensin homologue deleted on chromosome 10 (*PTEN*) genotyping also have important effects on the prognosis and treatment of gliomas (14). Tan et al. (35) used a radiomics nomograph to predict *IDH* genotyping (AUC: 0.900), confirming that most WHO LGG patients presented with *IDH* mutation and had better prognosis. Kanazawa et al. (36) found that ADC kurtosis (AUC: 0.728) and T2 kurtosis (AUC: 0.866) had the highest correlation with *1p/19q* codeletion through the radiomics texture analysis. In *MGMT* prediction, Li et al. (37) compared two different feature selection methods, among which the all-relevant features have the potential of offering better prediction power than the univariately-predictive and non-redundant features (AUC: 0.880). Based on multicenter and multimodal MRIs, Li et al. (38) illustrated that the radiomics features derived from T2WI were more correlated with *PTEN* genotyping (AUC: 0.787). Results from these studies suggest that radiomics analysis is indeed a powerful method for predicting glioma genotyping before surgery. Multimodal MRIs could further improve the prediction performance of glioma genotyping.

Multimodal MRI technology can obtain a variety of tumor information including tumor morphology, blood perfusion, and metabolism that can help further evaluate tumor prognosis and therapeutic effects. Several studies have explored the molecular-biomarkers-based classification of glioma subtypes using PET, DWI, DCE, and DSC-PWI, which are non-standard imaging modalities used to gather additional tumor information. Song et al. (39) demonstrated that both PET and DSC-PWI might be non-invasive predictors for *IDH* genotyping, in which PET combined with CBV could improve the differentiation of *IDH*-mutant astrocytoma and *IDH*-wildtype glioblastoma (AUC: 0.903). Kim et al. (40) found that DWI and PWI can improve the diagnostic performance of *IDH* genotyping (AUC: 0.747) to further guide the LGG glioma subtyping, with DWI-ADC features playing a significant role. Furthermore, Yan et al. (17) showed that multimodel-MRIs–based radiomics may be useful for noninvasive detection of molecular groups and guiding glioma subtyping. The image fusion model (multivariate logistic regression) incorporating radiomic features from T1CE and DWI-ADC achieved an AUC of 0.884 and 0.669 for predicting *IDH* and *TERT* status, respectively. Pei et al. (41) have also investigated the integration of multimodal MRIs to improve the accuracy of glioma subclassification. Adding DSC-PWI to conventional MRIs can improve glioma subtype prediction in patients with diffuse gliomas (AUC: 0.864, 0.787, and 0.816 in *IDH* wildtype, *IDH* mutant and *1p/19q*-noncodeleted, and *IDH* mutant and *1p/19q*-codeleted, respectively). In summary, the utilization of non-standard imaging techniques and machine-learning in glioma subtyping has exhibited encouraging outcomes. As the research in this area advances, it is essential to emphasize the reproducibility and generalizability of these methods to facilitate their potential incorporation into routine clinical practice. Furthermore, additional comprehensive studies are necessary to explore the effects of integrating imaging features with multimodal imaging data, with the aim of improving the accuracy and comprehensiveness of glioma subtyping.

*IDH* mutant tumors can produce oncometabolite 2-hydroxyglutarate (2HG) which can be non-invasively detected by *in vivo* MR spectroscopy (MRS). Studies have shown that elevated levels of 2HG can be detected in *IDH* mutant tumors using MRS, allowing for non-invasive assessment and confirmation of *IDH* mutation status (42, 43). Furthermore, the quantification of 2HG levels through MRS has demonstrated prognostic significance. Higher levels of 2HG in *IDH* mutant tumors have been associated with better treatment response and improved overall survival outcomes (44). Currently, radiomics methods and 2HG MRS are both non-invasive techniques used in the detection of *IDH* mutant tumors. The choice between these approaches depends on the specific clinical context, resource availability, and the required information for patient management.

In general, the radiomic method is superior to the 2HG MRS analysis manifesting in heterogeneity assessment, availability, generalizability, and predictive modeling for glioma *IDH* genotyping (45–47). *IDH* mutant gliomas often exhibit significant intratumoral heterogeneity, with diverse regions of aggressiveness, therapy resistance, and molecular characteristics. Radiomics methods can capture this heterogeneity by analyzing multiple regions within the tumor, allowing for a more comprehensive quantitative evaluation of the *IDH* mutant gliomas. However, 2HG MRS typically provides a global assessment of 2HG concentration throughout the entire tumor, potentially missing significant spatial variances. The majority of clinical MRI scanners are already equipped with imaging protocols essential for radiomics analysis. However, performing 2HG MRS often necessitates specific acquisition sequences and dedicated post-processing techniques, which may not be universally accessible in all clinical settings. Currently, radiomics methods are highly adaptable and enable the integration of multimodal imaging data, which can enhance diagnostic and predictive capabilities for *IDH* genotyping. In contrast, 2HG MRS is specific to MRI and solely focuses on measuring 2HG concentration. Radiomics methods often employ machine-learning modeling to extract valuable information from imaging data. By training models on large datasets, radiomics–based approaches not only have the ability to generate predictive models for *IDH* genotyping but also to guide comprehensive prognostic analysis for patients. This capability extends beyond the direct measurement of 2HG, providing a more comprehensive assessment of the tumor and its behavior.

In conventional radiomics analysis, some studies have analyzed the prediction potential of different machine-learning models for specific clinical tasks. Parmer et al. (48) compared 12 machine-learning models in terms of their prediction performance in patients with lung cancer, with random forest achieving the best result (AUC: 0.660 ± 0.030, RSD_AUC_: 4.5%). In another study about head and neck cancer, Parmer et al. (49) evaluated 11 machine-learning models in terms of their prediction performance of overall survival, with naive Bayes managing the highest prognostic performance (AUC: 0.670 ± 0.076, RSD_AUC_: 11.3%). Fang et al. (33) compared three machine-learning models in predicting *TERT* genotyping of LGGs, including random forest (AUC: 0.827 ± 0.043, RSD_AUC_: 5.2%), adaboost (AUC: 0.820 ± 0.040, RSD_AUC_: 4.9%) and linear SVM (AUC: 0.840 ± 0.090, RSD_AUC_: 10.7%). In our study, we compared the predictive effectiveness of eight machine-learning classifiers by using only CR features as model predictors, with LDA exhibiting the best prediction performance and stability (AUC: 0.833 ± 0.062, RSD_AUC_: 7.4%). Overall, the prediction performance of classifiers using only CR features is generally low, and the predictive ability of the same classifier varies greatly across different clinical tasks. Conventional radiomics analysis always depends on a fixed feature extraction pipeline, which could limit the predictive potential of machine-learning models.

To improve the predictive potential of machine-learning models, extracting features with abundant representational information is the key. CR features extracted by conventional radiomics are predefined and limited in number, resulting in the limited acquisition of tumor heterogeneities. However, a deep-learning model with end-to-end prediction capability can automatically extract features from each layer or transform and represent features layer by layer to obtain a variety of complex features that are closely related to tumor heterogeneities. In medical imaging analysis, some studies have shown that DL models have a powerful ability to improve the understanding of tumor characteristics. When Li et al. (23) were constructing the hierarchical *IDH* and *1p/19q* prediction models, they confirmed that DL features had better stability and reproducibility than CR features, with better generalization ability in *IDH* genotyping prediction (AUC: 0.85-0.89). Chen et al. (21) confirmed that DL features tended to outperform CR features, and the prediction performance of the DL model after adding CR features was improved (AUC: 0.910) in *PTEN* genotyping. Compared to CR features, DL features are extracted more flexibly and can provide more comprehensive information for the construction of machine-learning models. However, when considering the mixture of DL and CR features as predictors, few studies have analyzed the prediction effectiveness of different machine-learning models, and the optimal machine-learning model has not yet been determined for IDH genotyping prediction.

Therefore, in our study, we dug deeper into the information contained in MR imaging data and extracted two groups of features (CR and DL) for *IDH* genotyping. We analyzed and compared eight common machine-learning models aiming to find the optimal classifier. Features were extracted from different MRI modalities and tumor subregions, respectively. MRIs involved in this study were collected from our medical center and the public database TCGA, which may help to build a robust and broadly applicable model for *IDH* genotyping. Moreover, the region of interest in most studies was delineated by radiology specialists. However, we utilized a fully-automated segmentation approach to delineate glioma areas which were the focused zones of feature calculation. Compared to manual segmentation, the fully-automated segmentation methods can reduce the variation of delineation between different observers, produce more reproducible and stable features, and remain low-cost and time-saving options.

In recent studies, deep-learning models used for glioma genotyping were mainly from the CNN family. Chang et al. (50) developed a 2D-ResNet model to non-invasively predict *IDH* genotyping using conventional MR imaging data (AUC: 0.950). Li et al. (23) creatively built a novel 2.5D-ResNet18 model by designing an image sequence as independent input (AUC: 0.890). Some studies also extracted DL features directly from 3D glioma volumes. Chen et al. (21) integrated a 3D-ResNet model and CR features to collaboratively predict *PTEN* genotyping (AUC: 0.910). In our study, we constructed a 2.5D-CNN model to extract initial features from the image sequence and then used LSTM to learn the initial features in an ensemble way. Unlike the 2D- or 3D-CNN model, the 2.5D-CNN model can ensure access to rich tumor information and prevent model underfitting in a small cohort.

In our study for CR features selection, we used LASSO to reduce feature dimensionality and eliminate the risk of model overfitting caused by excessive features. In total, we selected the seven most valuable and stable CR features from ten feature subsets (**Supplementary Table S3**). Each feature had distinctive prediction potential in *IDH* genotyping (P<0.05). We found that the final selected CR features were primarily texture information, reflecting the characteristics of slow or periodic changes throughout the tumor. It is difficult to observe these tumor texture features with the naked human eye in imaging data. Quantitative analysis of the texture features will be an effective way to help clinicians understand and treat tumors.

Metrics, such as accuracy, specificity, and sensitivity, need to set a threshold in advance to determine whether the predictive sample is positive or negative. However, AUC, an indicator used to evaluate the classifier’s ability to distinguish samples between positive and negative, will not be affected by threshold adjustment. Therefore, we mainly refer to the AUC value to compare the prediction performance of classifiers (51). When we used a mixture of DL signatures and CR features as predictors, r-SVM had the highest accuracy but its stability was the worst (AUC: 0.911 ± 0.073, accuracy: 0.856 ± 0.056, RSD_AUC_: 8.0%), while LR had the best AUC and stability (AUC: 0.920 ± 0.043, accuracy: 0.843 ± 0.044, RSD_AUC_: 4.7%). If only the predefined CR features are used as the LR predictors, the learning ability of LR will be greatly limited and prediction performance will not be satisfactory (AUC: 0.830 ± 0.066, accuracy: 0.771 ± 0.051, RSD_AUC_: 8.0%). Compared with CR features, DL signatures exhibit superior prediction values (AUC: 0.915 ± 0.054, accuracy: 0.835 ± 0.061, RSD_AUC_: 5.9%). After adding CR features, the prediction performance of the DL–based model does not exhibit a significant improvement (AUC: 0.920 ± 0.043, accuracy: 0.843 ± 0.044, RSD_AUC_: 4.7%). Additionally, we confirmed that multimodal signatures improved the prediction performance of *IDH* genotyping and outperformed any single-modality signature in the comparison of prediction potentials. Among the single-modality signature, T1CE and T2WI had the best prediction results (T1CE, AUC: 0.904 ± 0.044, RSD_AUC_: 4.9%; T2WI, AUC: 0.899 ± 0.062, RSD_AUC_: 6.9%), considering as the important modalities in *IDH* genotyping. Furthermore, the t-SNE visualization suggested that the DL signatures possessed better discriminative ability compared to the CR features. Overall, the results from our analyses suggested that LR should be a preferable machine-learning classifier in *IDH* genotyping and DL signatures exhibit superior prediction values and discriminative capability.

The application of deep-learning for glioma genotyping is an inevitable trend in the future, but it is still in the initial stage and has certain limitations. Firstly, meeting the large sample size requirements of deep-learning is still a problem. A larger sample size and independent validation data are still required to assess the generalization of our model. Secondly, the study of multiple genotyping predictions will help us further understand the tumor characteristics and efficiently guide patients’ treatment. Thirdly, the biological mechanisms and clinical interpretations of how DL features relate to *IDH* genotyping still remain unclear. Although we illustrated that DL features have promising prediction value in *IDH* genotyping, further research and understanding are required.

## 5 Conclusion

Our findings highlight the clinical utility of deep-learning–based radiomics analysis for *IDH* genotyping. Through a nested 10-fold cross-validation process, we developed an efficient LR model with robust performance by combining CR features and DL signatures as model predictors. Through subgroup analysis, it is observed that DL signatures consistently outperform CR features in terms of prediction performance and discriminative capability. The addition of CR features does not significantly enhance the prediction performance of DL-signature–based model, indicating that DL signatures alone exhibit favorable prediction capability, with potential as a standalone approach for accurate predictions. Through t-SNE cluster analysis, DL signatures also display markedly superior clustering and discriminative capability in comparison to CR features. Overall, the future direction of radiomics analysis may revolve around the utilization of custom deep-learning features, emphasizing the importance of incorporating deep-learning techniques to extract robust and informative features from medical imaging data.

## Data availability statement

Data of the descriptive analysis, group comparison, and figure description are provided in the supplement. Additional data on the individual case level can be requested from the corresponding author. Requests to access these datasets should be directed to liuyun@njmu.edu.cn.

## Ethics statement

The studies involving humans were approved by The First Affiliated Hospital of Nanjing Medical University (2021-SR-098). The studies were conducted in accordance with the local legislation and institutional requirements. Written informed consent for participation was not required from the participants or the participants’ legal guardians/next of kin in accordance with the national legislation and the institutional requirements.

## Author contributions

HZ, JW and WF conceived and designed the study. YoY, JZ, XF, ZW, JN, and FY collected the molecular pathology and imaging data. HZ and JW performed image pre-processing and tumor segmentation. GZ, CW, YH and XZ analyzed the data and performed the statistical analysis. HZ, YuY, and YL wrote the manuscript. All authors contributed to the article and approved the submitted version.
