# Supplementary material for: Deep-learning and conventional radiomics to predict IDH genotyping status based on magnetic resonance imaging data in adult diffuse glioma
Source: Front Oncol. 2023 Aug 30;13:1143688. doi: 10.3389/fonc.2023.1143688 (PMC10499353; doi:10.3389/fonc.2023.1143688)
Supplement: Supplementary file 1 [file DataSheet_1.pdf]

Supplementary Material

Deep-Learning and Conventional Radiomics to Predict *IDH* Genotyping Status based on Magnetic Resonance Imaging Data in Adult Diffuse Glioma

Table S1 Data division in cross-validation

Loop	Training Cohort							Validation Cohort							P_{inter}
	Public			Local			P_{intra}	Public			Local			P_{intra}	
	Mutation	Wildtype	Total	Mutation	Wildtype	Total		Mutation	Wildtype	Total	Mutation	Wildtype	Total		
Loop1	83	112	195	96	146	242	0.61	6	17	23	14	12	26	0.09	1.00
Loop2	78	116	194	101	142	243	0.85	11	13	24	9	16	25	0.68	1.00
Loop3	81	117	198	98	141	239	1.0	8	12	20	12	17	29	1.0	1.00
Loop4	80	119	199	99	139	238	0.84	9	10	19	11	19	30	0.66	1.00
Loop5	81	112	193	98	146	244	0.78	8	17	25	12	12	24	0.32	1.00
Loop6	81	117	198	98	141	239	1.0	8	12	20	12	17	29	1.0	1.00
Loop7	79	117	196	100	141	241	0.88	10	12	22	10	17	27	0.76	1.00
Loop8	82	118	200	97	141	238	1.0	7	11	18	13	17	30	1.0	1.00
Loop9	80	118	198	99	141	240	0.93	9	11	20	11	17	28	0.92	1.00
Loop10	76	115	191	104	144	248	0.72	13	14	27	6	14	20	0.34	1.00

The P-values of IDH status are calculated between the public and local cohorts (P_{intra}) and between training and validation cohorts (P_{inter}) by using chi-square test.

Table S2 the description of eight classifiers

**Acronym**	**Machine-learning classifier name**	**Short description**
LR	logistic regression	A generalized linear regression model with the form $Y = WX + b$ (1).
kNN	k-nearest neighbors	A non-parametric learning algorithm that determines the class of the unknown sample based on the types of the nearest k samples (2).
NB	naive bayes	A classifier that is based on Bayes' theorem (3).
l-SVM	support vector machines with linear kernel	A machine-learning algorithm that is the specific support vector machine using the linear kernel (4).
r-SVM	support vector machines with radial basis function kernel	A machine-learning algorithm that is the specific support vector machine using the radial basis function (5).
RF	random forest	An ensemble classifier that uses multiple decision trees to train and predict samples (6).
Adaboost	adaptive boosting	A machine-learning algorithm that trains different weak classifiers on the same training set and then assembles them to create a strong classifier (7).
LDA	linear discriminant analysis	A machine-learning algorithm that projects high-dimensional sample features into the best discrimination vector space for classification (8).

Table S3 LASSO feature selection in each outer loop

**Loop**	**Feature Name**	**Number**
Loop 1	['flair_wt_original_shape_Sphericity', 'flair_wt_wavelet-HLL_firstorder_Skewness', 'flair_wt_wavelet-HLH_gldm_DependenceVariance', 'flair_tc_wavelet-LHL_firstorder_Skewness', 't1_wt_original_glrlm_RunEntropy', 't1_wt_wavelet-LLH_glrlm_RunEntropy', 't2_wt_wavelet-HLH_gldm_DependenceVariance', 't2_tc_wavelet-LLL_firstorder_10Percentile']	8
Loop 2	['flair_wt_original_shape_Sphericity', 'flair_tc_wavelet-LHL_firstorder_Skewness', 't1_wt_original_glrlm_RunEntropy', 't1_wt_wavelet-LLH_glrlm_RunEntropy', 't1ce_wt_wavelet-LHL_glszm_LargeAreaLowGrayLevelEmphasis', 't2_wt_wavelet-HLL_glcm_Idn', 't2_tc_wavelet-LLL_firstorder_10Percentile']	7

Loop 3	['flair_wt_original_shape_Sphericity', 'flair_wt_wavelet-HLH_gldm_DependenceVariance', 't1_wt_original_glrlm_RunEntropy', 't1_wt_original_gldm_DependenceEntropy', 't1_wt_wavelet-LLH_glrlm_RunEntropy', 't1ce_wt_wavelet-LLH_glcm_Imc1', 't2_wt_wavelet-HLL_glcm_Idn', 't2_wt_wavelet-LLL_glcm_Imc2', 't2_tc_wavelet-LLL_firstorder_10Percentile']	9
Loop 4	['flair_wt_wavelet-HLH_gldm_DependenceVariance', 'flair_wt_wavelet-HHL_glcm_DifferenceVariance', 'flair_wt_wavelet-HHL_glcm_SumSquares', 'flair_tc_wavelet-LHL_firstorder_Skewness', 't1_wt_original_glrlm_RunEntropy', 't1_wt_original_gldm_DependenceEntropy', 't2_tc_wavelet-LLL_firstorder_10Percentile']	7
Loop 5	['flair_wt_original_shape_Sphericity', 'flair_wt_wavelet-HLH_gldm_DependenceVariance', 'flair_tc_wavelet-LHL_firstorder_Skewness', 't1_wt_original_glrlm_RunEntropy', 't1ce_tc_wavelet-LLH_glszm_LargeAreaLowGrayLevelEmphasis', 't2_wt_wavelet-HLL_glcm_Idn', 't2_wt_wavelet-LLL_glcm_Imc2', 't2_tc_wavelet-LLL_firstorder_10Percentile']	8
Loop 6	['flair_wt_original_shape_Sphericity', 'flair_wt_wavelet-HLH_gldm_DependenceVariance', 't1_wt_original_glrlm_RunEntropy', 't2_wt_wavelet-HLH_gldm_DependenceVariance', 't2_wt_wavelet-LLL_glcm_Imc2', 't2_tc_wavelet-LLL_firstorder_10Percentile']	6
Loop 7	['flair_wt_original_shape_Sphericity', 'flair_wt_wavelet-HLH_gldm_DependenceVariance', 'flair_tc_wavelet-LHL_firstorder_Skewness', 't1_wt_original_glrlm_RunEntropy', 't1_wt_wavelet-LLH_glrlm_RunEntropy', 't2_tc_wavelet-LLL_firstorder_10Percentile']	6
Loop 8	['flair_wt_wavelet-HLH_gldm_DependenceVariance', 'flair_tc_wavelet-LHL_firstorder_Skewness', 't1_wt_original_glrlm_RunEntropy', 't2_wt_wavelet-LLL_glcm_Imc2', 't2_tc_wavelet-LLL_firstorder_10Percentile']	5
Loop 9	['flair_wt_original_shape_Sphericity', 'flair_wt_wavelet-HLH_gldm_DependenceVariance', 'flair_tc_wavelet-LHL_firstorder_Skewness', 't1_wt_original_glrlm_RunEntropy', 't2_wt_wavelet-HLL_glcm_Idn', 't2_tc_wavelet-LLL_firstorder_10Percentile']	6
Loop 10	['flair_wt_wavelet-HLH_gldm_DependenceVariance', 'flair_tc_wavelet-LHL_firstorder_Skewness', 't1_wt_original_glrlm_RunEntropy', 't1_wt_original_gldm_DependenceEntropy', 't1_wt_wavelet-LLH_glrlm_RunEntropy', 't2_wt_wavelet-HLL_glcm_Idn', 't2_tc_wavelet-LLL_firstorder_10Percentile']	7

Feature name consists of the description of the modality, tumor subregion, filter and feature type.

Table S4 the DL signatures and CR features used as predictors of the classifiers

Feature Name	Selected Time	P*
T1WI_signature		5.91e-69
T1CE_signature		1.03e-80

T2WI_signature		8.96e-78
FLAIR_signature		5.62e-66
T1WI _WT_original_glrlm_RunEntropy	10	4.11e-13
T2WI _TC_wavelet-LLL_firstorder_10Percentile	10	5.57e-22
FLAIR _WT_wavelet-HLH_gldm_DependenceVariance	9	7.53e-07
FLAIR _TC_wavelet-LHL_firstorder_Skewness	8	3.94e-04
FLAIR _WT_original_shape_Sphericity	7	1.18e-08
T2WI _WT_wavelet-HLL_glcm_Idn	5	2.76e-07
T1WI _WT_wavelet-LLH_glrlm_RunEntropy	5	1.87e-04

Feature names consist of the descriptions of the modality, tumor subregion, filter, and feature type. *P-values of features are calculated between IDH mutation and IDH wildtype by using unpaired *t*-test.

Table S5 the validation performance based on the different feature subcategories

Subcategory	Classifier	Accuracy	AUC	Specificity	Sensitivity	RSD_{AUC}
DL+CR	LR	0.843±0.044	**0.920±0.043**	0.896±0.036	0.769±0.109	**4.7%**
	kNN	0.848±0.065	0.904±0.081	**0.910±0.065**	0.758±0.107	9.0%
	NB	0.850±0.052	0.916±0.063	0.885±0.049	**0.799±0.092**	6.9%
	l-SVM	0.821±0.050	0.812±0.052	0.865±0.091	0.758±0.115	6.4%
	r-SVM	**0.856±0.056**	0.911±0.073	0.896±0.054	**0.799±0.092**	8.0%
	RF	0.842±0.051	0.917±0.052	0.882±0.067	0.783±0.090	5.7%
	Adaboost	0.827±0.050	0.903±0.044	0.864±0.066	0.773±0.091	4.9%
	LDA	0.839±0.048	0.918±0.045	0.885±0.036	0.774±0.114	4.9%
	Average	0.841±0.051	0.900±0.065	0.885±0.059	0.777±0.098	7.2%
DL	LR	0.835±0.061	**0.915±0.054**	0.882±0.046	0.769±0.129	5.9%
	kNN	**0.854±0.047**	0.908±0.057	**0.885±0.054**	**0.809±0.071**	6.3%
	NB	0.841±0.067	0.911±0.056	0.882±0.054	0.784±0.121	6.1%
	l-SVM	0.837±0.044	0.832±0.048	0.861±0.074	0.804±0.110	5.8%
	r-SVM	0.825±0.055	0.913±0.052	0.864±0.044	0.769±0.111	**5.7%**
	RF	0.829±0.054	0.911±0.056	0.857±0.063	0.788±0.083	6.1%
	Adaboost	0.829±0.062	0.900±0.054	0.854±0.081	0.794±0.073	6.0%
	LDA	0.827±0.068	0.913±0.055	0.875±0.056	0.759±0.124	6.0%
	Average	0.835±0.056	0.900±0.058	0.870±0.059	0.784±0.102	6.4%
CR	LR	0.771±0.051	0.830±0.066	0.833±0.076	0.683±0.081	8.0%
	kNN	0.772±0.050	0.824±0.085	0.906±0.054	0.577±0.107	10.3%
	NB	0.767±0.044	0.815±0.072	**0.913±0.055**	0.557±0.094	8.8%
	l-SVM	0.688±0.098	0.672±0.095	0.757±0.149	0.588±0.141	14.1%
	r-SVM	0.757±0.062	0.814±0.083	0.865±0.083	0.602±0.134	10.2%
	RF	0.763±0.090	0.813±0.099	0.809±0.094	**0.697±0.149**	12.2%
	Adaboost	0.736±0.083	0.791±0.104	0.833±0.091	0.597±0.141	13.1%
	LDA	**0.780±0.061**	**0.833±0.062**	0.872±0.084	0.647±0.123	**7.4%**
	Average	0.754±0.072	0.799±0.095	0.848±0.099	0.618±0.127	11.9%

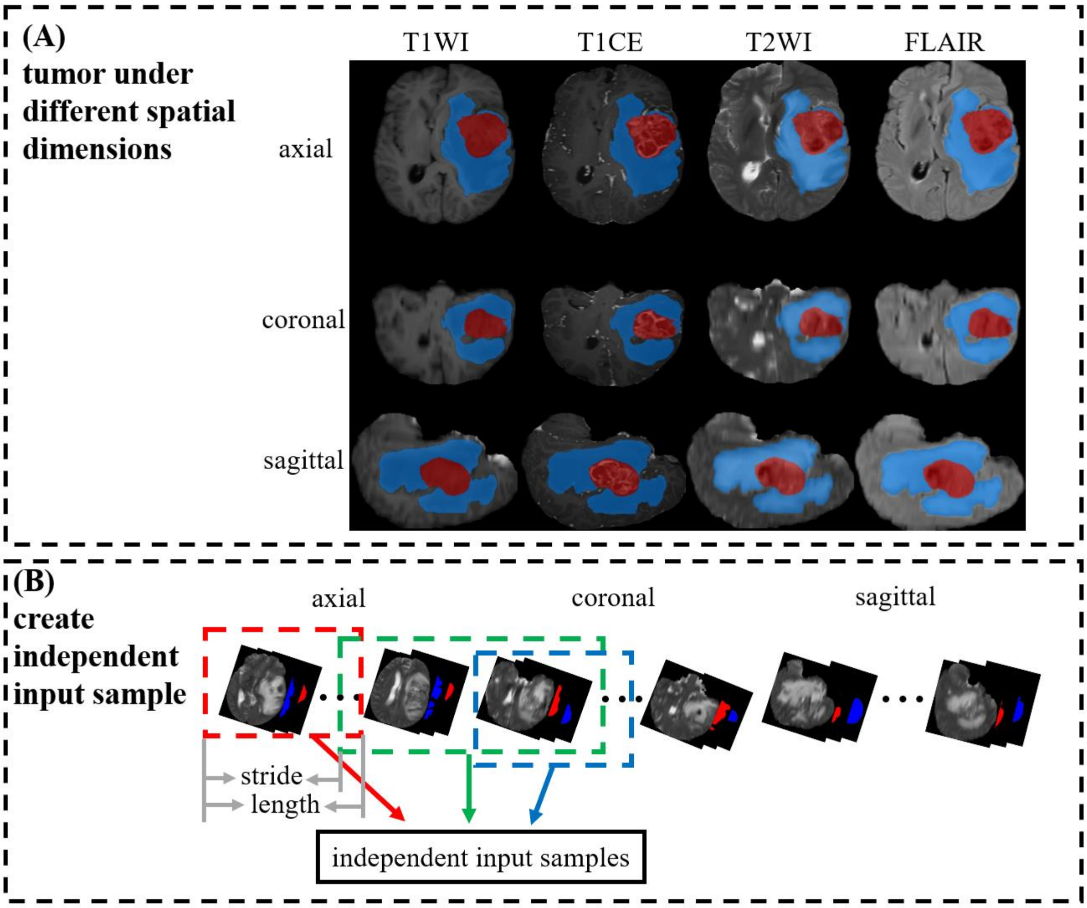

Figure S1. Data preparation for deep-learning model. (A) Tumor under different spatial dimensions. (B) Create independent input sample process. Splitting independent input samples from the 3-channel image sequence with a total length of 33 by setting the sample-sequence length and moving stride.

REFERENCES

1. Chen H, Zhang X, Wang X, Quan X, Deng Y, Lu M, Wei Q, Ye Q, Zhou Q, Xiang Z, et al. MRI-based radiomics signature for pretreatment prediction of pathological response to neoadjuvant chemotherapy in osteosarcoma: a multicenter study. *Eur Radiol* (2021) 31:7913–7924. doi: 10.1007/s00330-021-07748-6
2. Guo G, Wang H, Bell D, Bi Y, Greer K. KNN Model-Based Approach in Classification. In: Meersman R, Tari Z, Schmidt DC, editors. *On The Move to Meaningful Internet Systems 2003: CoopIS, DOA, and ODBASE*. Lecture Notes in Computer Science. Berlin, Heidelberg: Springer (2003). p. 986–996 doi: 10.1007/978-3-540-39964-3_62
3. Rish I. An empirical study of the naive Bayes classifier. *IJCAI 2001 workshop on empirical methods in artificial intelligence*. (2001). p. 41–46
4. Du J-Z, Lu W-G, Wu X-H, Dong J-Y, Zuo W-M. L-SVM: A radius-margin-based SVM algorithm with LogDet regularization. *Expert Systems with Applications* (2018) 102:113–125. doi: 10.1016/j.eswa.2018.02.006
5. Zhang X, Lu X, Shi Q, Xu X, Leung HE, Harris LN, Iglehart JD, Miron A, Liu JS, Wong WH. Recursive SVM feature selection and sample classification for mass-spectrometry and microarray data. *BMC Bioinformatics* (2006) 7:197. doi: 10.1186/1471-2105-7-197
6. Liaw A, Wiener M. Classification and regression by randomForest. *R news* (2002) 2:18–22.
7. Chen P, Pan C. Diabetes classification model based on boosting algorithms. *BMC Bioinformatics* (2018) 19:109. doi: 10.1186/s12859-018-2090-9
8. Hastie T, Tibshirani R, Buja A. Flexible Discriminant Analysis by Optimal Scoring. *Journal of the American Statistical Association* (1994) 89:1255–1270. doi: 10.1080/01621459.1994.10476866
